# Supplementary material for: Association Between Body Image and Quality of Life of Women Who Underwent Breast Cancer Surgery
Source: Int J Environ Res Public Health. 2025 Jul 15;22(7):1114. doi: 10.3390/ijerph22071114 (PMC12294835; doi:10.3390/ijerph22071114)
Supplement: Supplementary file 1 [file ijerph-22-01114-s001.zip › ijerph-3668342-supplementary.pdf]

Association between body image and quality of life of women who underwent breast cancer surgery

C.Z. Battistello, E. Remor, Í. M. Costa, M. E. de Oliveira and A. P. S. Damin

Supplementary material

Table S1 presents descriptive statistics (i.e., Min.-Max., Mean, SD) for the current sample's quality of life (FACT-B) and perceived body image (BIRS) scores.

Table S1. Descriptive statistics for FACT-B and BIRS in women Brazilian sample who underwent breast cancer surgery (N = 106).

Variables	Min.	Max.	Mean	SD
Functional assessment of Cancer Therapy for Breast Cancer (FACT-B) [b]				
Physical well-being [b]	3.00	24.00	17.01	5.12
Family social well-being [b]	0.00	28.00	19.95	5.71
Emotional well-being [b]	5.00	24.00	17.47	4.94
Functional well-being [b]	5.00	28.00	18.46	5.37
Additional Wellbeing Concerns [b]	6.00	38.00	23.91	7.23
General quality of life [FACT-B Total score] [b]	35.00	134.00	96.82	21.05
Body Image and Relationships Scale (BIRS) [a]				
Strength and health [a]	12.00	55.00	35.22	10.06
Social barriers [a]	9.00	45.00	24.43	8.76
Appearance and sexuality [a]	11.00	51.00	30.54	8.09
General body image [Total score] [a]	38.00	140.00	90.19	21.86

a = Higher scores on this scale indicate worse self-evaluation, impairment, or decline in body image; b = Higher scores indicate a better-perceived quality of life.

Tables S2 and S3 present the associations of sociodemographic characteristics with perceived body image (BIRS, total score) and quality of life (FACT-B, total score) scores. In addition, S2 presents the associations of cancer staging with perceived body image (BIRS) and quality of life (FACT-B) scores.

Table S2. Associations of age, cancer staging with perceived body image (BIRS), and quality of life (FACT-B) scores (N = 106).

	**Coefficient correlation, (p-value)**		
Psychological scores	**Age**	**Time since diagnosis**	**Cancer staging (Grade 1 to 4)**
General body image [Total score] (BIRS) [a]	**-0.230 (0.020)[1]**	-0.020 (0.839)[3]	0.145 (0.193)[3]
Strength and Health (BIRS) [a]	-0.183 (0.065)[2]	0.004 (0.971)[3]	0.175 (0.113)[3]
Social Barriers (BIRS) [a]	**-0.209 (0.035)[2]**	-0.062 (0.533)[3]	0.145 (0.194)[3]
Appearance and Sexuality (BIRS) [a]	-0.168 (0.089)[2]	0.039 (0.695)[3]	0.019 (0.864)[3]
General quality of life [FACT-B total score] [b]	**0.314 (0.001)[1]**	0.001 (0.989)[3]	-0.121 (0.277)[3]
Physical well-being (FACT-B) [b]	**0.195 (0.005)[1]**	0.010 (0.923)[3]	-0.125 (0.259)[3]
Family social well-being (FACT-B) [b]	0.107 (0.124)[1]	-0.023 (0.814)[3]	0.027 (0.807)[3]
Emotional well-being (FACT-B) [b]	**0.220 (0.002)[1]**	0.001 (0.993)[3]	-0.087 (0.435)[3]
Functional well-being (FACT-B) [b]	**0.321 (<.001)[1]**	0.089 (0.367)[3]	-0.048 (0.664)[3]
Additional Wellbeing Concerns (FACT-B) [b]	0.133 (0.180)[1]	-0.046 (0.645)[3]	-0.097 (0.382)[3]

a = Higher scores on this scale indicate worse self-evaluation, impairment, or decline in body image; b = Higher scores indicate a better-perceived quality of life; 1 = Pearson correlation 2 = Kendall correlation; 3 = Spearman correlation

Table S3. Descriptive statistics of the psychological scores of the sociodemographic groups compared (N = 106).

**Sociodemographic variables according to questionnaires' scores.**	**Group categories**	**Mean**	**SD**	**p-value**
Have religion (BIRS)[a]	No	91.00	16.98	0.913
	Yes	89.98	22.25	
Have religion (FACT-B)[a]	No	97.33	19.99	0.962
	Yes	96.90	21.28	
Have kids (BIRS)[a]	No	85.12	24.42	0.316
	Yes	91.10	21.39	
Have kids (FACT-B)[a]	No	100.75	17.63	0.420
	Yes	96.12	21.61	
Married (BIRS)[a]	No	89.06	20.46	0.644
	Yes	91.06	23.03	
Married (FACT-B)[a]	No	98.30	20.94	0.538
	Yes	95.68	21.23	
Occupation (BIRS)[b]	Formal work	93.18	21.64	0.052
	Non-formal work	90.69	21.33	
	Retired	78.05	20.34	
Occupation (FACT-B)[b]	Formal work	94.93	21.11	0.355
	Non-formal work	97.41	19.65	
	Retired	103.17	22.63	

a = T-test, b = ANOVA with Tukey’s post hoc test

Table S4 presents the association of health characteristics with perceived body image (BIRS) or quality of life (FACT-B) scores.

Table S4. Descriptive statistics of psychological scores of the compared groups (alcohol and tobacco use, psychiatric medication, psychological support, chemotherapy) (N = 106).

Variables	Domains	Yes (M; SD; Mdn)	No (M; SD; Mdn)	Statistics	p-value	ES
Alcohol and tobacco use (yes; n = 17; no; n =89)	SB (BIRS)	30.74; 9,55; 32	23.17; 8,19; 22	U = 375,000	0.001	-0.32*
Psychiatric medications (yes; n = 31; no; n =75)	PWB (FACT-B)	15.51; 5.28; 17	17.64; 4.96; 19	U = 879,500	0.049	-0.19*
	EWB (FACT-B)	15.61; 4.81; 16	18.24; 4.82; 19	U = 786,500	0.009	-0.25*
	FWB (FACT-B)	16.51; 5.67; 17	19.26; 5.06; 20	U = 816,000	0.016	-0.23*
	AC (FACT-B)	21.74; 6.18; 21	24.81; 7.48; 25	t(104) = 2.01	0.046	0.43**
Received psychological counseling (yes; n = 39; no; n = 67)	AS (BIRS)	32.89; 8.40; 31	29.17; 7.64; 29	t(104)=-2.32	0.002	0.53**
Chemotherapy (yes; n = 63; no; n =43)	SH (BIRS)	37.35; 9.99; 38	32.06; 9.53; 35	t(104)=-2.75	0.007	0.55**
	SB (BIRS)	27.16; 8.73; 27	20.51; 7.27; 19	U = 754,500	0.000	0.37*
	General body image [Total score] (BIRS)	95.59; 20.98; 97	82.39; 20.94; 83	t(104)=-3.17	0.002	0.63**

BIRS: SB = Social Barriers, AS = Appearance and Sexuality, SH = Strength and Health; FACT-B: PWB = Physical Well-being, EWB = Emotional Well-Being, FWB = Functional Wellbeing, AC = Additional concerns; M = Mean; SD = Standard Deviation; Mdn = Median; U = Mann–Whitney U test: t = Student's t-test; * = Cohen’s *r* for non- parametric tests; ** = Cohen's *d* for parametric tests; ES = Effect size;

Table S5 compares (non-parametric tests) perceived body image (BIRS) and quality of life (FACT-B) scores by the type of surgery.

Table S5. Descriptive statistics of psychological scores of the compared groups (Type of Surgery) (N = 106).

Psychological scores	Type of surgery [median (IQR)]			Conservative vs. Mastectomy	Conservative vs. Mastectomy vs. Reconstruction
	Conservative (n = 69)	Mastectomy (n = 26)	Reconstruction (n =11)	U-Mann Witney Statistics (p-value)	Kruskal Wallis Test Statistics (p-value)
General body image [Total score] (BIRS) [a]	93 (29.5)	92 (27.2)	91 (36.5)	834.00 (0.672)	0.413 (0.813)
Strength and Health (BIRS) [a]	37 (14)	35 (11.2)	36 (11.5)	895.50 (0.990)	0.589 (0.745)
Social Barriers (BIRS) [a]	23.5 (12.2)	24 (14.2)	22 (10.5)	816.50 (0.586)	0.335 (0.864)
Appearance and Sexuality (BIRS) [a]	29 (11)	29.5 (13.2)	36 (12)	831.00 (0.581)	0.574 (0.750)
General quality of life [FACT-B total score] [b]	98 (29)	97.5 (32.7)	90 (25.5)	881.00 (0.894)	0.913 (0.634)
Physical well-being (FACT-B) [b]	19 (6)	17 (8.5)	16 (4)	727.00 (0.155)	3.266 (0.195)
Family social well-being (FACT-B) [b]	21 (7)	21.5 (6)	20 (4)	859.50 (0.754)	0.237 (0.888)
Emotional well-being (FACT-B) [b]	18 (7)	19 (8.7)	17 (8)	877.50 (0.870)	0.136 (0.937)
Functional well-being (FACT-B) [b]	19 (6)	19.5 (6.7)	17 (6.5)	834.50 (0.601)	3.098 (0.212)
Additional Wellbeing Concerns (FACT-B) [b]	24 (11)	25.5 (10.5)	26 (5.5)	872.50 (0.838)	0.058 (0.971)

a = Higher scores on this scale indicate worse self-evaluation, impairment, or decline in body image; b = Higher scores indicate a better-perceived quality of life. IQR = Interquartile rage
